# Identifying the Molecular Mechanisms and Types of Cell Death Induced by *bio*- and *pyr*-Silica Nanoparticles in Endothelial Cells

**DOI:** 10.3390/ijms23095103

**Published:** 2022-05-04

**Authors:** Katarzyna Solarska-Ściuk, Kinga Adach, Mateusz Fijałkowski, Katarzyna Haczkiewicz-Leśniak, Michał Kulus, Mateusz Olbromski, Natalia Glatzel-Plucińska, Oskar Szelest, Dorota Bonarska-Kujawa

**Affiliations:** 1 Department of Physics and Biophysics, Wrocław University of Environmental and Life Sciences, Norwida St. 25, 50-375 Wroclaw, Poland; dorota.bonarska-kujawa@upwr.edu.pl; 2 Department of Advanced Materials, Institute for Nanomaterials, Advanced Technologies and Innovation, Technical University of Liberec, 461 17 Liberec, Czech Republic; kinga.adach@tul.cz (K.A.); mateusz.fijalkowski@tul.cz (M.F.); 3 Division of Ultrastructural Research, Faculty of Medicine, Wroclaw Medical University, Chalubinskiego 6a, 50-368 Wroclaw, Poland; katarzyna.haczkiewicz@umw.edu.pl (K.H.-L.); michal.kulus@umw.edu.pl (M.K.); 4 Department of Histology and Embryology, Wroclaw Medical University, Chalubinskiego 6a, 50-368 Wroclaw, Poland; mateusz.olbromski@umw.edu.pl (M.O.); natalia.glatzel-plucinska@umw.edu.pl (N.G.-P.); 5 ICLab, Wyżynna 8H, 30-617 Cracow, Poland; oskar@irtech.pl

**Keywords:** silica nanoparticles, oxidative stress, cell death, cell migration, HMEC-1

## Abstract

The term “nanosilica” refers to materials containing ultrafine particles. They have gained a rapid increase in popularity in a variety of applications and in numerous aspects of human life. Due to their unique physicochemical properties, SiO_2_ nanoparticles have attracted significant attention in the field of biomedicine. This study aimed to elucidate the mechanism underlying the cellular response to stress which is induced by the exposure of cells to both biogenic and pyrogenic silica nanoparticles and which may lead to their death. Both TEM and fluorescence microscopy investigations confirmed molecular changes in cells after treatment with silica nanoparticles. The cytotoxic activity of the compounds and intracellular RNS were determined in relation to HMEC-1 cells using the fluorimetric method. Apoptosis was quantified by microscopic assessment and by flow cytometry. Furthermore, the impact of nanosilica on cell migration and cell cycle arrest were determined. The obtained results compared the biological effects of mesoporous silica nanoparticles extracted from *Urtica dioica* L. and pyrogenic material and indicated that both types of NPs have an impact on RNS production causing apoptosis, necrosis, and autophagy. Although mesoporous silica nanoparticles did not cause cell cycle arrest, at the concentration of 50 μg/mL and higher they could disturb redox balance and stimulate cell migration.

## 1. Introduction

Nanotechnology is a dynamically developing branch of science that focuses on a wide spectrum of nanoparticles and their possible applications in a multitude of disciplines. This science contributes to the significant development of new therapies which are based on the applications of nanoparticles. In the multiplicity of the types of nanoparticles, there are also studies focused on silica nanoparticles (SiNPs). The main feature of mesoporous silica nanoparticles is their numerous pores that are suitable for loading cargo and transporting drugs/biomarkers into the cells and parts of the organism.

The vast majority of the studies conducted were based on nanoparticles which were obtained for SiNPs greater than 50 nm [1]. By contrast, in the present work, we are concentrated on the impact of mesoporous silica nanoparticles which have a size grain smaller than 20 nm, namely, biogenic silica nanoparticles from stinging nettle and pyrogenic silica, synthetically produced, on endothelial cells [2]. Numerous studies on silica nanoparticles related to cytotoxicity effects have been conducted in relation to erythrocytes, endothelial cells, and different types of cancer cells [3]. The results obtained proved that SiO_2_ nanoparticles are responsible for changes in the shape of erythrocytes and are also able to induce hemolysis in erythrocytes, and that they decreased the survival of HMEC-1 cells, enhanced reactive oxygen species production [2], and induced cytotoxicity and apoptosis in both human skin epithelial (A431) and human lung epithelial (A549) cells [3].

It is a well-known fact that intravenously injected nanoparticles initially interact with the inner linings of the lumen blood vessels. Therefore, nanosilica may have an impact on vascular homeostasis, as well as the maintenance of their function. In order to maintain a cellular redox balance, it is necessary to investigate the impact of oxidative stress induced by reactive oxygen (ROS) and nitrogen species (RNS) on cells. There are only a few studies of the size-dependent toxicity on endothelial cells of silica nanomaterials smaller than 50 nm in size [1]. On the one hand, their physico-chemical properties and dosage can induce the production of both ROS and RNS, which, in turn, initiate signaling pathways leading to cell death (apoptosis, necrosis, or autophagy). On the other hand, the cytotoxic effect may have a therapeutic value, which means that silica nanoparticles might be exploited in the treatment of multiple diseases due to the mechanism of cell death modulation, which may be used as a new therapeutic pathway [4].

The most common form of cell death is apoptosis (namely, a programmed cell death), which is characterized by depolymerization of the cytoskeleton, cell shrinkage, chromatin condensation, inter-nucleosomal DNA fragmentation, the formation of small vesicles named apoptotic bodies, as well as the transportation of phosphatidylserine to the cell surface [5,6,7].

Necrosis is the next method of cell death. It is caused by either external and physical factors (e.g., low or high temperature, radiation, ultraviolet radiation) or mechanical factors. This could contribute to the inhibition of metabolism, a decrease in ATP level, electron transport disturbances, as well as the inflow of water and ions inside the cell. Finally, in the late stages of necrosis, this process leads to swelling of the organelle and the whole cell, digestion of cell structure elements (by enzymes released from bursting lysosomes), and, as a consequence, the breakdown and release of its contents into the extracellular space [4,8].

The autophagy process is extremely important for maintaining cell viability/survival under stress conditions by selectively removing damaged cells, cell organelles, and protein aggregates [9]. Autophagy is an adaptive mechanism, and it is also considered an important lysosome-based pathway of necrosis. This process is characterized by the degradation of the components of the cell structure directly inside it by autophagic vacuoles [10,11].

Silica nanoparticles are among the most widely used nanomaterials, demonstrating the highest effectiveness due to their low level of toxicity and small particle size, which enable nanoparticles to enter into the blood circulation and be used for drug delivery, bioimaging, and in another treatment protocols [5]. However, it has been proved that silica nanoparticles are safe only at appropriate dosages [2] due to the fact that long-term exposure to low-dose or short-term exposure to higher-dose nanoparticles can lead to the poisoning of the organism. This is the main reason why the usage of silica nanomaterials in the development of nanotechnology needs monitoring of their toxicity and biological effects in order to ensure safe biomedical application [12]. Oxidative stress has an indirect impact on physiological functions. One of these is its impact on the cell cycle and on cell migration, specifically for endothelial cells (an increase in the level of oxidative stress leads to stimulation of the cell migration) [13,14]. Whereas silica nanoparticles induce oxidative stress and are internalized by endocytosis, they are also free in the cytoplasm. After being taken up into endothelial cells, they cause disruption and disorganization of F-actin fibers [15]. They are responsible for causing morphological changes in cells, mitochondrial membrane damage, autophagy, the downregulation of cell adhesion protein internalization [16], delays in the S and G2/M phases of the cell cycle, and DNA damage in the human hepatocyte cell line HL-7702 [5].

In this study, the HMEC-1 cell line was used as a model system to explore the properties of biogenic and pyrogenic silica nanoparticles in the process of the death of human dermal microvascular endothelial cells and in order to investigate the mechanisms involved. Increasing concentrations of both *bio*SiO_2_ and *pyr*SiO_2_ resulted in an increased degree of cell death, which was determined to occur via apoptosis, necrosis, and autophagy (depending on the type of nanoparticles). The effects of nanosilica exposure on cell cycle arrest, cell migration, and the level of RNS were checked. Nanoparticles which were extracted from *Urtica dioica* L. presented slightly higher toxicity than pyrogenic nanoparticles in HMEC-1. What is more, TEM confirmed the internalization of silica nanoparticles into the studied cell line. Condensed chromatin and fragmented nuclei were proved using a fluorescence microscope and a transmission electron microscope. Although images obtained through the stimulated emission depletion technique proved that the concentration of 50 μg/mL (after 24 h incubation) for both tested types of silica nanoparticles is safe for cells and does not have a significant effect on the cytoskeleton, the fluorescence of the actin cytoskeleton was noticeably more intense in cells treated with SiO_2_ compared to control.

The data obtained presents the importance of the type of nanoparticles, concentration, and the time of exposure in the processes of cells interactions. The study shows that *bio*-nanoparticles present good compatibility at the appropriate dosage, a fact which makes them a promising material in therapeutics.

## 2. Results

### 2.1. Transmission Electron Microscope (TEM)

**Figure 1:**
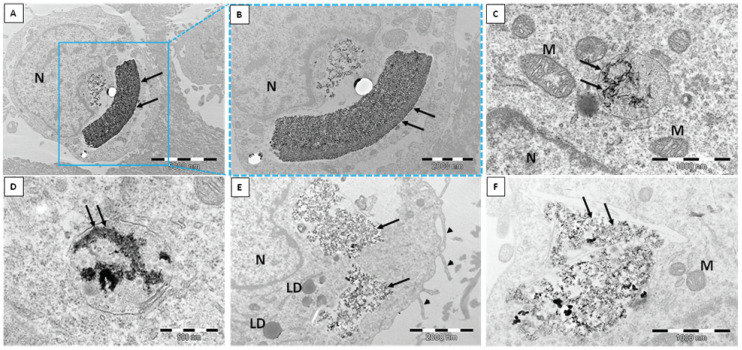
Internalization of silica nanoparticles into the HMEC-1 cell line. Large and electron-dense aggregates of *bio*SiO_2_ (**A**,**B**) and more dispersed nanoparticles of *pyr*SiO_2_ (**E**,**F**) in the cytoplasm or inside the vesicles. Arrows—SiO_2_ nanoparticles; N—nucleus; M—mitochondria with lamellar cristae; LD—lipid droplets; arrowheads—cell membrane cytoplasmic extensions. (**A**–**D**)—*bio*SiO_2_; (**E**,**F**)—*pyr*SiO_2_.

**Figure 2:**
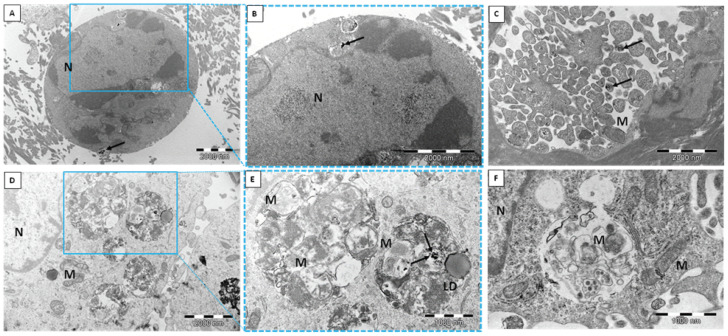
Apoptosis and autophagy of HMEC-1 cell line after exposure to silica nanoparticles. (**A**,**B**)—cell membrane smoothing; cytoplasm condensation and chromatin rearrangement are clearly visible. (**C**)—apoptotic bodies. (**D**–**F**)—the autophagolysosomes with different, partially digested, contents. Arrows—SiO_2_ nanoparticles; N—nucleus, LD—lipid droplets; M—mitochondria. (**A**,**B**,**D**,**E**)—*pyr*SiO_2_; (**C**,**F**)—*bio*SiO_2_.

**Figure 3:**
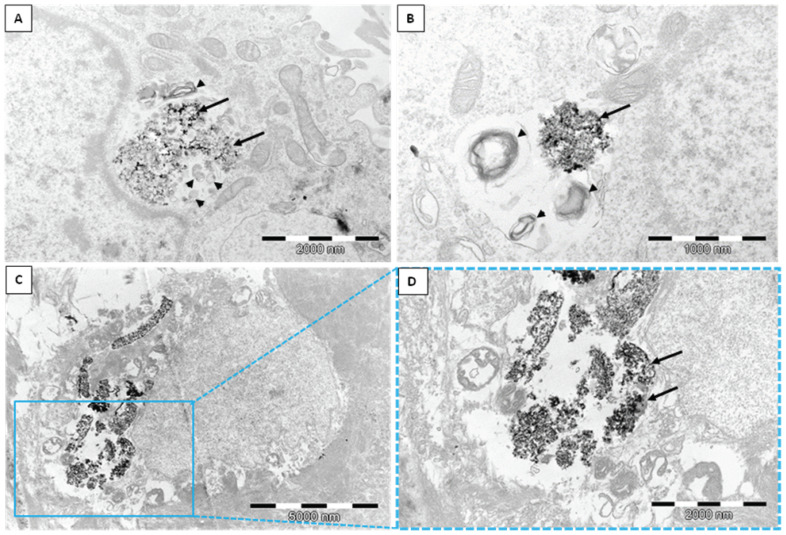
Autophagy and necrosis of HMEC-1 cell line after exposure to silica nanoparticles. (**A**,**B**)—silica nanoparticles internalized into autophagolysosomes (arrows). Cellular debris is also visible inside the autophagolysosomes (arrowheads). (**C**,**D**)—cell necrosis after internalization of *bio*SiO_2_. The disruption of cytoplasmic constituents is conspicuous.

**Figure 4:**
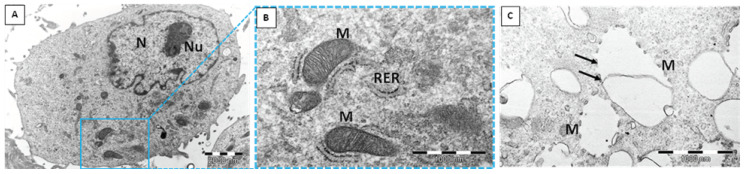
Control group cells. Proper ultrastructure without signs of cell death is distinct (**A**,**B**). For comparison, degenerated mitochondria after exposure to silica nanoparticles are depicted on (**C**). Arrows—remnants of damaged mitochondrial membrane. N—nucleus; Nu—nucleolus; M—mitochondria; RER—rough endoplasmic reticulum.

The human dermal microvascular endothelial cell line (HMEC-1) was exposed to two different types of silica nanoparticles, namely, biogenic (*bio*SiO_2_) and pyrogenic (*pyr*SiO_2_) silica at a concentration of 50 μg/mL for 72 h. TEM confirmed the internalization of silica nanoparticles into the studied cell line (Figure 1). Despite internalization in a similar manner, there were numerous differences in the silica itself. Biogenic silica created more compact and extremely large aggregates, while pyrogenic silica formed small aggregates (Figure 1A–F). Moreover, *bio*SiO_2_ was much more electron-dense than *pyr*SiO_2_ in TEM. Both biogenic and pyrogenic types were internalized directly into the cytoplasm (Figure 1A,B) or both types were found in vesicles separated by either single or multiple membranes (Figure 1C–F). The HMEC-1 cells with biogenic and pyrogenic silica had many features in common. Almost all cells contained on their surface many cytoplasmic processes of the cell membrane. The characteristic features also included an abundant rough endoplasmic reticulum (RER), numerous polyribosomes, an extensive Golgi apparatus, lipid droplets, centrioles, and numerous mitochondria with lamellar as well as tubular cristae. In addition, there were highly lobulated nuclei with interchromatin and perichromatin granules, and islets of heterochromatin close to the nuclear envelope, all of which were visible. Both types of silica nanoparticles induced pathological changes in the cell ultrastructure; one of the most prominent features was a large number of swollen and degenerated mitochondria in the form of myelin structures and dilation of the Golgi apparatus cisternae. Furthermore, three types of cell death with different ultrastructural features, namely, apoptosis, autophagy, and necrosis, were evoked by silica nanoparticles to varying degrees and intensity. Apoptosis was observed in TEM at different stages. Condensed chromatin and cytoplasm were visible, as well as the apoptotic bodies (Figure 2 A–C). Moreover, *pyr*- and *bio*SiO_2_ induced the accumulation in the cytoplasm of large numbers of autophagosomes and autophagolysosomes containing flocculent digested cellular material, fragments of nuclei, digested mitochondria with blurry cristae, and lipid droplets (Figure 2D–F). Moreover, the studied silica was present in autophagolysosomes together with the other cellular components designed to be digested (Figure 3A,B). The cells internalizing silica also exhibited features of necrosis, including the disintegration of the cytoplasm and mitochondria, and ruptures of the cell membrane (Figure 3C,D). Compare to the experimental group of exposed cells, cells of the control group possessed normal mitochondria without edema with lamellar or tubular cristae, abundant RER, numerous heterochromatin clumps, and nucleoli with distinct granular and fibrillar components (Figure 4A,B).

### 2.2. Phalloidin Imaging Using STED Nanoscopy

**Figure 5:**
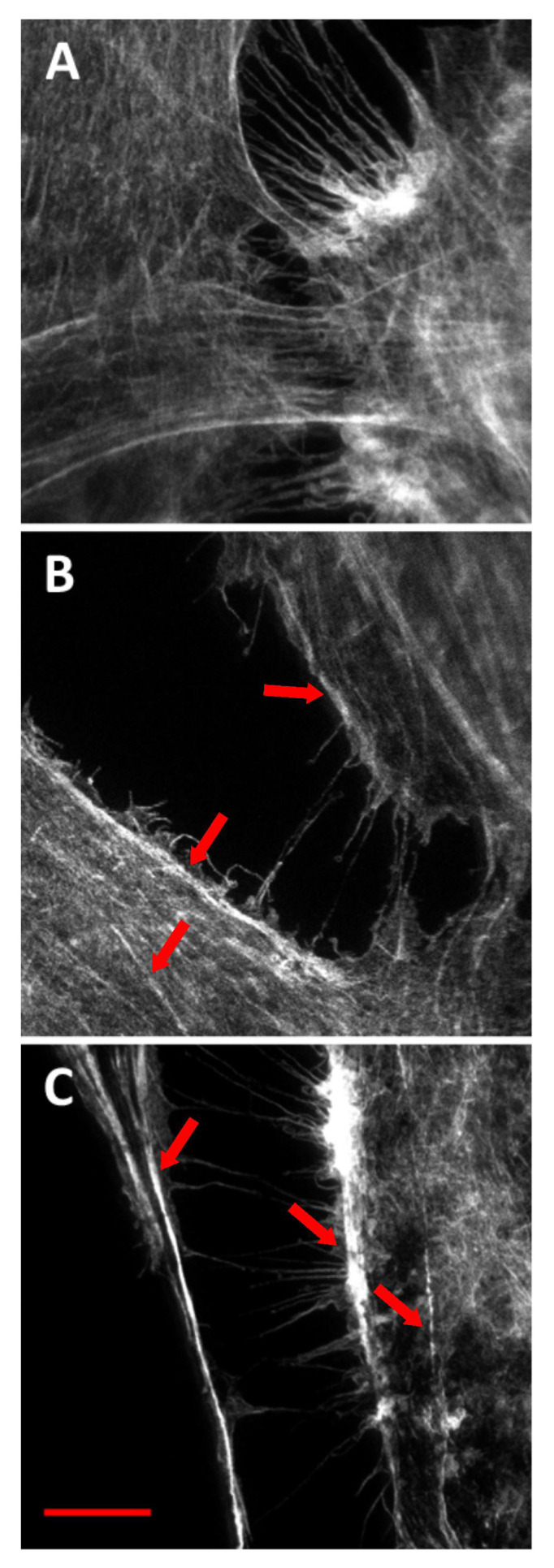
The actin cytoskeleton of HMEC-1 cells was assessed with phalloidin staining ((**A**)—control cells; (**B**)—cells after 24 h incubation with *bio*SiO_2_; (**C**)—cells after 24 h incubation with pyrSiO_2_). Samples were imaged using the STED technique. Scale bar: 5 μm. Red arrows show where phalloidin-positive actin stress fibers are present in the cell.

The actin cytoskeleton of human endothelial cells was assessed using phalloidin staining and STED microscopy focused on the ventral surface (Figure 5A–C). The images proved that the concentration of 50 μg/mL for both *bio*- and *pyr*-silica nanoparticles at the incubation period of 24 h is safe for cells. Furthermore, biogenic and pyrogenic nanosilica had no significant effects on the cytoskeleton; however, the fluorescence of the actin cytoskeleton was noticeably more intense in cells treated with silica nanoparticles than in those used as a control within the body of the cell and at the cell periphery (actin stress fibers were generally thicker).

### 2.3. Cell Migration Assay

**Figure 6:**
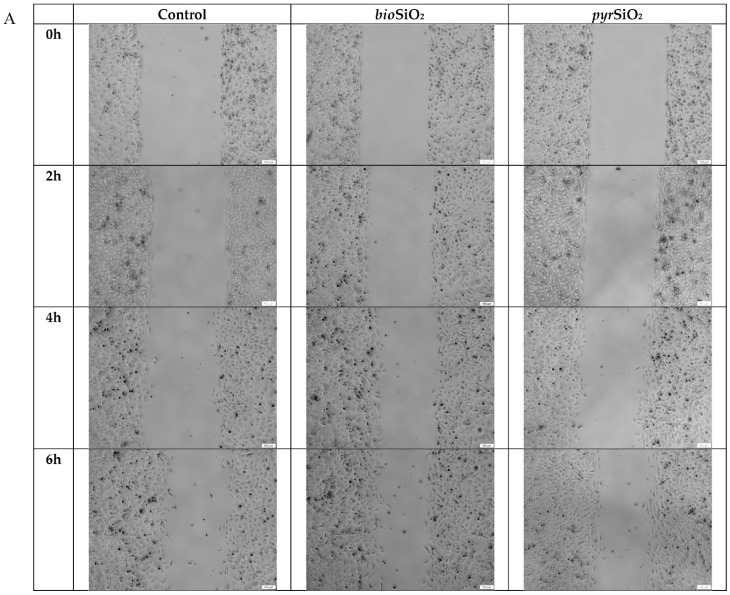

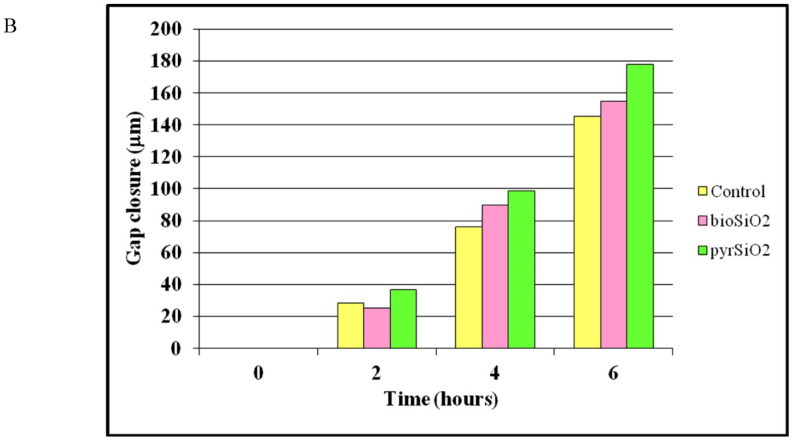
Impact of biogenic and pyrogenic silica nanoparticles on cell motility in vitro. (**A**)—the migration distance (the distance of the gap was measured in μm); (**B**)—the speed of the cell migration (relative migration = gap area at 0 h, 2 h, 4 h, or 6 h). V (speed) = S (road in μm)/t (time in h). Samples were imaged using a light microscope, 40×. Scale bar: 100 μm.

Cells treated with both tested silica nanoparticles showed an increase in the gap closure rate compared to the untreated control group. Based on the width of the gap (Figure 6A,B), the migration distance and the speed of the cell migration at 25.82 μm/h after treating with *bio*SiO_2_ and 29.61 μm/h after treating with *pyr*SiO_2_ were calculated. The speed of the cell migration for the control was 24.67 μm/h.

### 2.4. Microscopic Assessment of Apoptosis

**Figure 7:**
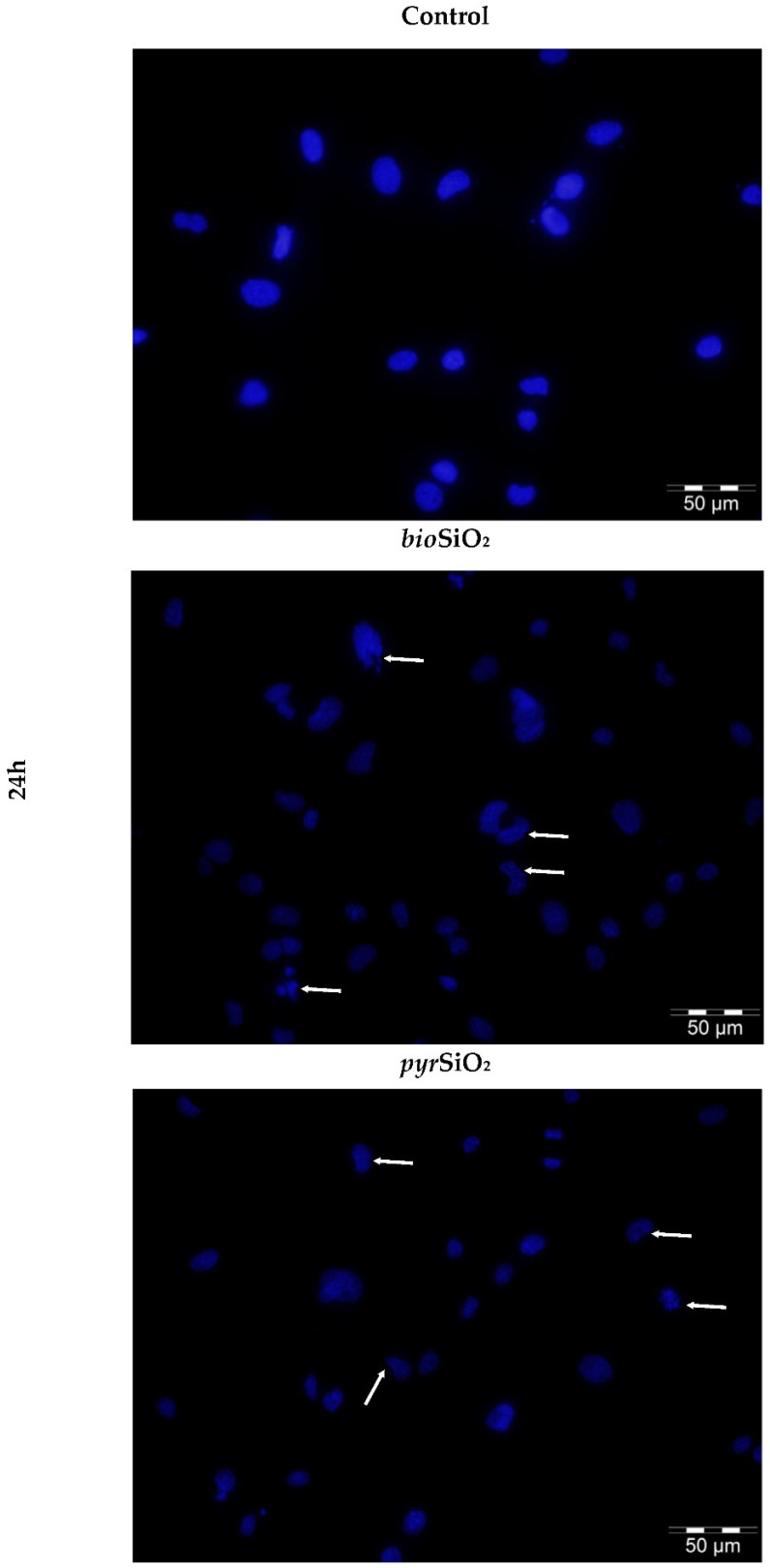
Cells incubated with silica nanoparticles for 24 h, seen stained for apoptosis with Hoechst 33258. Silica-nanoparticle-treatment-induced apoptosis-related morphological changes in the nuclei of HMEC-1, such as chromatin condensation and nuclear fragmentation, were marked with a white arrow (→). Olympus BX51 fluorescence microscope, 400×. Scale bar: 50 μm.

Figure 7 indicates impact effects exerted by biogenic and pyrogenic silica nanoparticles on the morphology of nuclei in endothelial cells, an observation which was proved using Hoechst 33258 staining. The nuclei of the untreated control group showed a uniform blue fluorescence. After adding *bio*- and *pyr*SiO_2_, the staining of the nuclei was dense and fragmented, and granular fluorescence was also observed. *Bio*SiO_2_ was a more potent inductor of apoptosis than *pyr*SiO_2_. Pyrogenic nanoparticles were responsible for many rapid morphological changes in the nuclei of HMEC-1, such as chromatin condensation and nuclear fragmentation.

### 2.5. Apoptosis and Cell Cycle Assay

**Figure 8:**
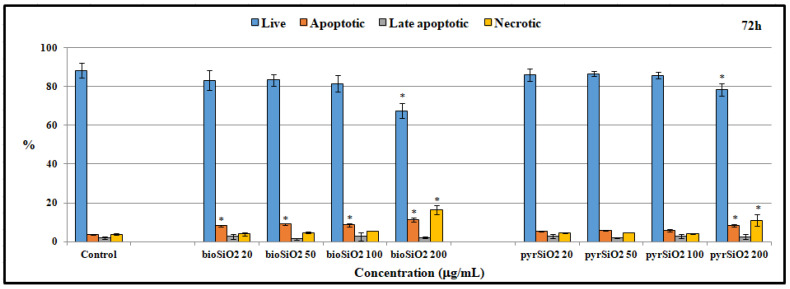
Occurrence of apoptosis, late apoptosis, and necrosis in cells with silica nanoparticles (0–200 μg/mL) after 72 h of incubation. Statistical evaluation of differences was conducted using the ANOVA I and Tukey’s post hoc tests, at significance levels of *p* < 0.01 (*) with respect to control.

In order to detect apoptotic and necrotic cells, dual staining with Annexin V-FITC and Propidium Iodide FITC Annexin V Apoptosis Detection Kit was used. This method is extremely common and sensitive due to the fact that the binding of Annexin V to phosphatidylserine is employed (this phospholipid is translocated into the plasma membrane from the internal to the external monolayer during the process of apoptosis). These results confirm that biogenic and pyrogenic silica nanoparticles induced apoptosis and necrosis in HMEC-1 cells. Apoptosis and necrosis were observed in both silica nanoparticles after 24, 48, and 72 h of incubation. This effect was dependent on the time of *bio*SiO_2_ and *pyr*SiO_2_ incubation and on silica concentration (Figure 8). Whereas the concentration and time of incubation increased in both nanoparticles, an increase in the fraction of apoptotic and necrotic cells was observed, mainly for *bio*SiO_2_ and after 72 h of incubation.

Furthermore, the flow cytometry analysis of the cell cycle after 24, 48, and 72 h of incubation showed that the nanoparticles used did not cause any statistical changes in its progression for *bio*SiO_2_. Therefore, we present results only for 72 h (Table 1). Untreated cells remained in a balanced distribution of the cell cycle. The collected data indicate a decrease in the level of cells in both the S and the G2/M cell cycle phases at the highest concentration of *pyr*SiO_2_. This may suggest that the nanoparticles caused excessive oxidative stress (a fact which was confirmed in a previous study) and that they are toxic for HMEC-1 cells.

### 2.6. RNS Production

**Figure 9:**
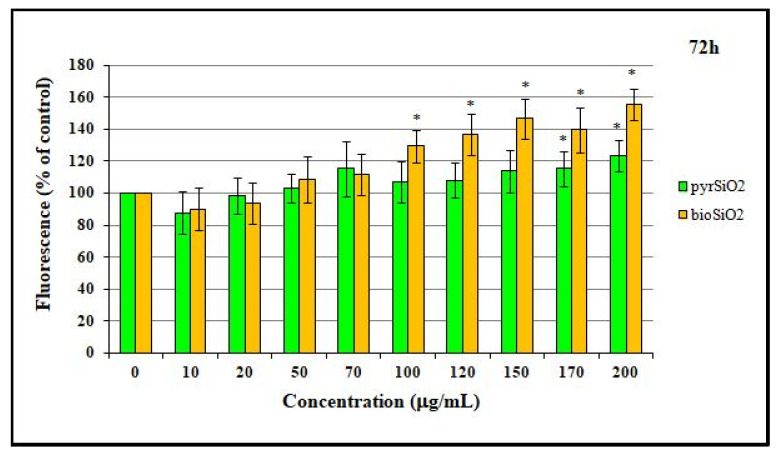
Intracellular reactive nitrogen species (RNS) generation in HMEC-1 cells. Production of RNS of human dermal microvascular endothelial cell line after treatment of silica nanoparticles (pyrogenic and biogenic). Statistical evaluation of differences was conducted using the ANOVA I and Tukey’s post hoc tests, at significance levels of *p* < 0.01 (*) with respect to the control.

After the incubation of cells with biogenic and pyrogenic SiO_2_ nanoparticles, the fluorescence intensity of the products of the oxidation of DAF-FM-DA was increased, depending on the nanoparticle concentration and the time of exposure (24, 48, and 72 h) with HMEC-1 cells. The effect was significant for the highest concentrations after 72 h of incubation for both tested silica nanoparticles, in particular for *bio*SiO_2_ (Figure 9). Moreover, at lower levels, namely, up to the concentration of 70 μg/mL, the differences between *bio*- and *pyr*-silica nanoparticles were not observed.

## 3. Discussion

In this study, the impact of biogenic and pyrogenic SiO_2_ nanoparticles on the biophysical properties of endothelial cells (HMEC-1) was analyzed. Several experiments showed that silica has a direct impact on the life/death of cells and the redox balance. Although the reactive oxygen species demonstrated a crucial role in cytotoxicity mechanisms, they also had an impact on apoptosis/necrosis/autophagy in various cell lines with silica nanoparticles. The complex process of the autophagy caused by SiO_2_-mediated cytotoxicity is still unclear [1].

The obtained results demonstrated the cellular response of *bio*- and *pyr*SiO_2_ nanoparticles, both of which were below 20 nm in size, within a broad range of concentrations from 0 up to 200 μg/mL. We showed that oxidative stress enhanced cell migration (Figure 6A,B) and was an important factor that contributed to the transformation from normal cells to angiogenesis. This was possible in human endothelial cells due to the fact that the vascular endothelial growth factor (VEGF) induced migration, which was stimulated via NAD(P)H oxidase (NOX)-derived reactive oxygen species production in a Rac1-dependent mechanism [13]. It is a well-known fact that one of the most important functions of endothelial cells is to form new vessels from pre-existing vasculature angiogenesis, a property which is significant in the case of many diseases, and it is also observed in normal physiology and wound healing [16]. Endothelial cell lines are inside blood vessels and regulate both blood pressure and its flow. This is possible through hemodynamic forces in the form of shear stress and mechanical strains which also act on the actin cytoskeleton, level of cell nitric oxide, and cell proliferation [17,18].

Nitric oxide (NO) is an important substance that is produced by the endothelium. This soluble gas is necessary in order to conduct vasodilatation and inflammation and to generate oxidative stress, mainly through the production of reactive oxygen species (ROS). Moreover, reducing the level of nitric oxide may facilitate vascular inflammation, which, in turn, leads to both the oxidation of lipoproteins and the formation of foam cells which are the precursor of atherosclerotic plaque. The consequences of disturbances in endothelial function are important in cardiovascular disease [19]. The study showed a beneficial impact of the tested nanoparticles on endothelial cells, as the changes in the level of RNS were observed only after 72 h of incubation together with an increase in concentrations (Figure 9). The obtained results confirmed that *bio*SiO_2_ nanoparticles had a more significant impact on the production of reactive nitrogen species, a fact which confirmed the previous results in which an increase in ROS (time- and concentration-dependence) for the same nanomaterials was proved. This suggests that silica nanoparticles (at high concentrations) induced oxidative stress, which promoted the production of NO—a process that was only mediated by mitochondria. Reactive oxygen species were formed in mitochondria, and this may explain many physiological processes (the regulation of autophagy, innate immunity or differentiation, the initiation of apoptosis through a release of cytochrome c, and the important role it plays in the regulation of homeostasis). Increasing the production of reactive oxygen species through the mitochondrial respiratory chain had a great impact on mitochondrial dysfunctions. This directly contributed to mitochondrial DNA damage and disturbances in oxygen consumption rates, which, in turn, had an impact on ROS production, further intensified oxidative stress, and aggravated damage within both mitochondria themselves and the entire cell [20]. Therefore, significant damage in mitochondria with blurry cristae and visible edema was observed. The TEM analysis confirmed either the internalization of silica nanoparticles into the HMEC-1 cell line directly into the cytoplasm or in vesicles separated by single or multiple membranes (Figure 1) compared to the control group (Figure 4). Both types of silica nanoparticles induced pathological changes in the cell ultrastructure, including the condensation of the cytoplasm and pyknotic nuclei, apoptotic bodies, large numbers of autophagosomes, autophagolysosomes, fragments of nuclei, and lipids droplets (Figure 2A–F and Figure 7).

A similar process was observed in the case of HUVEC, in which cells treated with silica nanoparticles displayed autophagic vacuoles with partially degraded cytoplasmic materials, as well as swollen and cristae-rupturing mitochondria. Duan et al. suggested that internalized silica nanoparticles could either attack mitochondria directly or cause mitochondrial damage indirectly via oxidative stress. Moreover, the F-actin in silica nanoparticles treated with HUVECs (at a high concentration of 100 μg/mL) was disorganized. The structure of F-actin was non-isotropically assembled and the fluorescent intensity of these fibers was weakened [15,21]. By comparison, although biogenic and pyrogenic nanosilica at a lower concentration (50 μg/mL) had no significant effect on the cytoskeleton (Figure 5), the fluorescence of the actin cytoskeleton was more intense in cells treated with silica nanoparticles, which were, in general, significantly thicker. The results presented by Yao et al. showed that brief exposure to a relatively low dosage of oxidative stress could enhance the stress fiber density in cells (in this case, in myoblasts). The opposite effect was noticed as a result of chronic exposure and a high dosage of oxidative stress, which led to a significant decrease in the stress fiber density, a reaction which could suppress the stress fiber density in cells [22,23,24]. Moreover, the oxidative-stress-mediated activation of protein tyrosine kinases could also impair adherent junctions, which suggests a loss in endothelial barrier integrity [25]. This was also confirmed in our previous study, in which the morphological changes of HMEC-1 became more and more obvious, in line with an increase in the concentration of silica nanoparticles. Moreover, cell density reduction, irregular shape, and cellular shrinkage were also observed. The cellular morphology changes were reflected in the viability of the cells [2]. The effects of biogenic and pyrogenic silica nanoparticles on the morphology of nuclei in endothelial cells were confirmed using both fluorescence microscopy and transmission electron microscopy. After incubating cells with *bio*- and *py*rSiO_2_ nanoparticles, the staining of the nuclei was dense and fragmented, and granular fluorescence was also observed (Figure 7). TEM analysis confirmed the presence of crescent-shaped chromatin typical of apoptosis (Figure 2A,B). The obtained result proved that biogenic nanoparticles were a more potent inductor of necrosis (Figure 3C,D) than pyrogenic nanoparticles. Moreover, these nanoparticles were able to cause oxidative damage in cellular membranes and organelles, namely, mitochondria, lysosomes, and the nucleus, which led to apoptosis or necrosis [4,26]. This was confirmed by Ye et al. [27], who reported that silicon dioxide (SiO_2_) regulated the expression of the *TP53* and *BAX* genes in nanoparticles in a dose-, size-, and exposure-time-dependent manner. Furthermore, silica nanoparticles showed that reactive-oxygen-species-mediated oxidative stress caused apoptosis in L-02 cells, a fact which confirmed that nanoparticles were able to have an impact on mitochondrial pathways [28]. In this study, apoptosis, necrosis, and autophagy were confirmed using several methods (fluorescence microscopy, TEM, and apoptosis assays). This study showed that the main form of death in the HMEC-1 cell line (after treatment with silica nanoparticles) is apoptosis (Figure 2). Although the main form of cell death is apoptosis, for the highest dosages of nanoparticles and the longest periods of incubation, necrosis was reported (Figure 3 and Figure 8). Moreover, autophagy (Figure 2 and Figure 3) was also observed after subjecting the tested cells to both *bio*- and *pyr*-nanoparticles. It is important to underline that the process of autophagy is an antiapoptotic mechanism which is focused on the protection of a cell by removing (due to the impact of reactive forms of oxygen) damaged organelles. Therefore, autophagy may be a mechanism for survival, and it does not necessarily lead to cell death. In the case analyzed here, the range of damage in a cell was greater than its ability to repair itself; therefore, autophagy may lead to cell death. It is our suggestion that the accumulation of SiO_2_ nanoparticles in liposomes leads to cell death, which is known as lysosomal cell death. Moreover, when the content of lysosomes is released into the cytosol, other forms of cell death can be activated, namely, apoptosis and necrosis [29]. The process of autophagy was also detected in the HMEC-1 cell line, which was found in HUVECs in which electron-dense SiNPs were internalized into the cells through endocytic pathways. In both our and other researchers’ works, silica nanoparticles were dispersed in the cytoplasm either freely or as membrane-bound aggregates in lysosomes or in swollen and cristae-rupturing mitochondria (Figure 3A–D) [15,21]. Although in this study no particles were found in the nuclei, Duan et al. reported that DNA damage measured by a comet assay showed in HUVECs with nanosilica (25, 50, 75, 100 μg/mL) that the DNA damage caused by silica nanoparticles was more serious and proportional to an increase in the dosages used [30]. Zhou et al. studied biological transmission using electron microscopy (which is usually adopted to observe the intracellular distribution of MSNs after endocytosis) and reported that present mesoporous silica nanoparticles were transported to large vesicular endosomes after being internalized and were then fused with lysosomes. Their results showed that the membranes of endosomes/lysosomes were eventually disrupted, a fact which suggested that nanosilica could escape from the endosomes/lysosomes; therefore, a large number of nanoparticles were observed in the cytoplasm that maintained their spherical morphology [31]. Thus, silica nanoparticles entering the cell through endocytosis might constitute one explanation for the observed decrease in membrane fluidity. Moreover, both nanoparticle-induced lipid peroxidation in order to alter the cell morphology and membrane roughness in human lymphocytes were reported [32,33]. Furthermore, the results indicated that 20 nm silica nanoparticles induced endothelial cell death either through the induction of reactive oxygen species, endoplasmic-reticulum-stress-mediated apoptotic cell death, or autophagy-mediated necrotic cell death or through the PI3K/AKT/eNOS signaling axis. Nevertheless, further studies in vivo are necessary to confirm that nanosilica with a diameter < 20 nm poses greater risks to cells in terms of cytotoxic effects [1,34]. In particular, a necrotic effect was reported after incubating HMEC-1 cell line with SiO_2_ nanoparticles at the highest concentration, i.e., 200 μg/mL. An analysis of the cell cycle (after 24, 48, and 72 h of incubation) showed that the used nanoparticles did not cause any statistical changes in its progression. Only in the case of the highest concentration of *pyr*SiO_2_ did the data indicate a decrease in the level of cells in both S and G2/M cell cycle phases, a fact which may be ascribed to the impact of excessive oxidative stress (Table 1). In other studies, the cell cycle (for the HUVEC cell line and silica nanoparticles with a spherical shape and an average diameter of 62 nm) was arrested at the G2/M phase, in which the percentage of cells at the G2/M phase increased progressively in a dose-dependent manner, which may have been triggered by oxidative DNA damage [30].

In summary, the presented data confirmed that both *bio*SiO_2_ and *pyr*SiO_2_ nanoparticles have a direct impact on the human dermal microvascular endothelial cell line (HMEC-1). This corroborates results from our previous studies in which we noticed that the safe concentration of silica nanoparticles for cells was equal to 50 µg/mL [2]. Although the FDA classifies silica as “generally recognized as safe”, its individual characteristics depend on a number of factors, namely, the time of exposure, the dosage used, and the type of silica nanoparticles. Although numerous in vitro studies have reported no toxicity for up to 100 μg/mL [35], the most important task for further research seems to be obtaining significant results in vivo regarding accumulating nanoparticles in organs (liver, spleen, or kidneys). All in all, mesoporous silica nanoparticles are an extremely promising material not only for cancer therapy (to kill cancer cells) but also for drug delivery and bioimaging in order to prevent dangerous and irreversible changes in the human body.

## 4. Materials and Methods

### 4.1. Silica Nanoparticles

For this study, we selected nettle (*Urtica dioica* L.), namely, a plant which has a high content of biogenic silica (*bio*SiO_2_). The method for obtaining the nanoparticles was presented in our previous study [2]. The pyrogenic silica (*pyr*SiO_2_), synthetically produced, was purchased from Sigma Aldrich (Poznań, Polska).

### 4.2. Cell Culture

A human dermal microvascular endothelial cell line (HMEC-1) was purchased from American Type Culture Collection (ATCC CRL 3243). The cells were cultured in a medium (MCDB 131) containing 10% FBS (fetal bovine serum), 10 mM L-glutamine, 1 μg/mL hydrocortisone, 1% penicillin/streptomycin, and 10 ng/mL EGF (epidermal growth factor), which were purchased from either Gibco or Sigma Aldrich and kept under 5% CO_2_ in plastic flasks at 37 °C. The cell density for seeding was chosen in order to assure logarithmic cell growth until the time for conducting an analysis.

### 4.3. Transmission Electron Microscopy Method (TEM)

To evaluate alterations in the ultrastructure of the HMEC-1 cell line after the internalizing of silicon dioxide SiO_2_, the TEM method was used. The endothelial cells were seeded in 100 mm cell culture dishes (1,250,000 per dish) with 10 mL of culture medium and incubated for 24 h. The cells were treated with biogenic and pyrogenic SiO_2_ at the concentration of 50 μg/mL (at 37 °C for 72 h). In the next step, the cells were carefully washed with 10 mL of PBS and trypsinized. After that, trypsin was neutralized with the culture medium and the cells were collected and centrifuged (800 rpm, 5 min, RT). Next, the cells were rinsed (twice) with PBS, centrifuged (800 rpm, 5 min, RT) and suspended in PBS. After passing through the process of centrifugation for 8 min at 1800 rpm, the pellets of cells were fixed (25 min at room temperature) in the 3.6% glutaraldehyde (Serva Electrophoresis, Heidelberg, Germany) diluted in 0.2 M cacodylate buffer with saccharose (Chempur, Piekary Śląskie, Poland). Subsequently, the fixative was rinsed (three times, 5 min) with the 0.1 M cacodylate buffer. Next, the samples were left in the same buffer at 4 °C overnight. The following day, one drop of bovine thrombin (Biomed, Lublin, Poland, 1 amp. with 400 a.u. lyophilized dissolved in 5 mL of PBS) and two drops of fibrinogen (1 mg/mL; Merck KGaA, Darmstadt, Germany) were added to each sample. The content was gently shaken until the cells were entrapped within the fibrin clot. The formed clots were divided into smaller parts and post-fixed in 1% (*w*/*v*) osmium tetroxide OsO_4_ (Serva Electrophoresis) prepared in cacodylate buffer (0.1 M) for 1 h at room temperature, followed by rinsing the samples with 0.1 M cacodylate buffer 3 × 5 min. The dehydration process was carried out at room temperature and for 10 min per step. Briefly, the specimens were passed through a series of graded ethanol concentrations (30%, 50%, 70%, Stanlab, Lublin, Poland) and left overnight at 4 °C in 70%. The next day, the clots were transferred to the solutions of 80% and 90% ethanol, then to a mixture of 90% ethanol/90% acetone (1:1). Afterward, the material was transferred through a series of graded acetone solutions (90%, 95%, and 100%). After dehydration, the material was incubated with a mixture of pure acetone and epoxy resin (Serva Electrophoresis) at ratios of 3:1 (20 min), 1:1 (60 min), and 1:3 (60 min), and finally transferred to a pure epoxy resin and incubated overnight at 4 °C. Then, the dehydrated and resin-saturated material was placed in the specimen boxes (flat embedding molds, Pelco, Ted Pella, Redding, CA, USA) and embedded in the epoxy resin with an accelerator 2,4,6-tris (dimethylaminomethyl) phenol in order to facilitate its polymerization. It was placed at 60 °C for 7 days.

In the first step, excessive resin was removed and the embedded clots of cells were trimmed and cut using ultramicrotome Power Tome XL (RMC, Tucson, AZ, USA) into semithin sections (600 nm thickness) with a Histo diamond knife (Diatome, Nidau, Switzerland).

The sections, dried on the heating plate at 100 °C, were subjected to differential staining with toluidine blue (Serva Electrophoresis) and sodium carbonate anhydrous (Alchem, Toruń, Poland) and closed with a Euparal mounting agent (Carl Roth, Mannheim, Germany). After staining the group of approximately 30 cells of the HMEC-1, one cell line was selected for making ultrathin, 70 nm-thick sections with the usage of Ultra 45° Diamond Knife (Diatome) for TEM documentation.

The ultrathin sections were collected onto the rhodium copper grids (Maxta form, 200 mesh, Ted Pella, Redding, CA, USA). The grids were stored on a petri dish for 24 h to allow for a strong adhesion process of the sections and then subjected to a double contrasting technique of ultrathin sections with uranyl acetate and lead citrate trihydrate (Serva Electrophoresis). The samples were visualized under TEM JEM-1011 (Jeol, Tokyo, Japan) operating at 80 kV. The electron micrographs were assembled using the TEM imaging platform iTEM1233 equipped with a Morada Camera (Olympus, Münster, Germany) at magnifications ranging from 3 to 200 K.

### 4.4. Phalloidin and STED Imaging

To visualize F-actin, the cells were stained with Abberior Star Red phalloidin 200 U/mL. HMEC-1 cells were seeded on Millicell EZ SLIDE 8-well glass culture slides (5000 cells per well) and cultured for 24 h. After that time, both 100 μL of fresh medium and 100 μL of medium with *bio*- and *pyr*-silica nanoparticles were added to the cells. In this experiment, nanoparticles at concentrations of 0, 20, 50, and 100 μg/mL were used (50 μg/mL is presented). The cells treated with nanoparticles were incubated under standard conditions. In the next step, the cells were rinsed twice with PBS and fixed with 4% paraformaldehyde in PBS for 12 min at room temperature, washed twice with PBS, and permeabilized with PBS—0.2% Triton-X 100 at room temperature for 10 min. Afterwards, the cells were washed and phalloidin (1:200) in blocking buffer (5% BSA, 0.2% Triton-X 100 in PBS) amounting to 50 μL per sample was added. The samples were incubated for 50 min at room temperature under humid conditions and in the dark. Next, the cells were rinsed 2–3 times with PBS 5 min per wash, and Fluoroshield with DAPI (an aqueous mounting medium for preserving the fluorescence of cell smears) was added. The samples were imaged using the STED (stimulated emission depletion) technique (Abberior Instruments GMBH, STEDyCON module installed on Nikon TI2E microscope body). The images were acquired with a 100× Nikon oil immersion objective (a 20 nm pixel size, a 775 nm pulsed depletion laser, and a 640 nm pulsed excitation laser with a 650–700 nm detection range).

### 4.5. Cell Migration Assay

Cell migration assays were carried out using a culture insert (ibidi culture insert 2 well, ibidi GmbH, Martinsried, Germany) at a density of 10,000 cells per well. After allowing the cells to attach overnight, the medium was removed and a fresh medium was added in order to control the cells, and medium with nanoparticles (at concentrations of 50 μg/mL) was added to the samples and incubated for 72 h. After that time, the culture insert was removed. The cells were washed with PBS and fresh medium was added to each sample. The plate was photographed at 0, 2, 4, 6, and 24 h to capture different fields at each time point on each plate. The number of cells that migrated was manually counted in three fields per well using a light microscope at 40× magnification. Based on the image analysis, we quantified the width of the gap filling rate (using the EPview software, Olympus), and calculated the migration distance as well as the speed of the cell migration at μm/h.

### 4.6. Microscopic Assessment of Apoptosis

Apoptotic cells (which are characterized morphologically by chromatin condensation and a fragmentation of nuclei) were quantified by staining with Hoechst 33258. Human endothelial cells were seeded on Millicell EZ SLIDE 8-well glass culture slides (5000 cells per well) and cultured for 24 h. Next, the cells were treated with *bio*SiO_2_ and *pyr*SiO_2_ 50 μg/mL) at 37 °C for 24 h. After the time of incubation, the cells were fixed by ice-cold 100% methanol for 10 min at −20 °C. Subsequently, the cells were quickly rinsed three times with PBS. Afterwards, Hoechst 33258 (2 μL of a 2 mg/mL stock solution) was added and the cells were incubated for 15 min in the dark at room temperature (RT). Then, they were rinsed twice with PBS buffer and observed using an Olympus BX51 fluorescence microscope (400×).

### 4.7. Flow Cytometry—Apoptosis and Cell Cycle Assay

The ability of the silica nanoparticles to induce apoptosis and cell cycle arrest was studied using flow cytometry analysis. The HMEC-1 cells were seeded in 6-well flat-bottom culture plates (25,000 per well) with 1 mL of culture medium and incubated for 24 h. Afterwards, the medium was removed and 1 mL of culture medium containing silica nanoparticles at appropriate concentrations (0, 20, 50, 100, and 200 μg/mL) was added to the well. Next, the plates were incubated at 37 °C under 5% CO_2_ for 24, 48, and 72 h. After incubation, the cells were carefully washed with 1 mL of PBS. Subsequently, the cells were trypsinized and suspended in the MCDB-131 medium. After that, they were washed twice with cold PBS and centrifuged (400× *g*, 7 min, RT). The cells were stained with a FITC Annexin V Apoptosis Detection Kit I, according to the manufacturer’s protocol. For each sample, data from a minimum of 10,000 events were collected. The results were further analyzed using the FlowJo 10.5 software (FlowJo, Asham, OR, USA).

For the next test—a cell cycle assay—the cells were suspended in a small amount of ice-cold PBS and fixed overnight at 4 °C in ice-cold 70% ethanol. Subsequently, the cells were stained by adding FxCycle PI/RNase staining solution (according to the manufacturer’s protocol). Next, the samples were incubated (30 min at 37 °C in the dark). The propidium iodide (PI) fluorescence was measured using a BD FACS Canto II flow cytometer (Becton Dickinson, Franklin Lakes, NJ, USA). In this experiment, for each sample, data from a minimum of 10,000 events were collected. The results were further analyzed using the ModFit LT 5.0 software (Verity Software House, Topsham, ME, USA). In both tests described here, the cell cycle analysis and apoptosis assay were performed in 3 independent replications.

### 4.8. RNS Production

Reactive nitrogen species production was estimated in HMEC-1 cells seeded on 96-well black plates (5000 cells per well) and cultured for 24 h. Next, silica nanoparticles were added at appropriate concentrations and the process of incubation was continued for another 24, 48, and 72 h at 37 °C under 5% CO_2_. Afterwards, the cell monolayers were rinsed with HBSS, and in the following step a fluorogenic probe, 5 μM of 3-amino-4-aminomethyl 2′,7′-dichlorofluorescein diacetate (DAF-FM-DA) in HBSS, was added. DAF-FM-DA is hydrolyzed to DAF-FM, which becomes fluorescent upon reaction with nitric oxide and its oxidation products. Directly after 2 h of incubation in the dark at 37 °C under 5% CO_2_, the fluorescence was read at λ_ex_ = 485 nm and λ_em_ = 510 nm.

After that, the DNA content was determined. For this purpose, after measuring RNS, the cells were frozen at −70 °C. Subsequently, the cells were thawed, 100 μL of distilled water per well was added, and the cells were re-frozen. Next, the cells were thawed again. Later, 50 μL of 15 μg/mL ribonuclease A was added to each well and incubated for 30 min in the dark at 37 °C. Afterwards, 50 μL of 10 μM propidium iodide in deionized water was added to the cells and incubated for 15 min under the same conditions. Finally, the fluorescence intensity of PI was measured at λ_ex_ = 355 nm and λ_em_ = 620 nm. The rate of RNS generation was calculated according to the following formula:

Rate of RNS = intensity of DAF-FM-DA fluorescence/intensity of PI fluorescence.

The value of the fluorescence in the control samples was assumed to be 100%.

## 5. Conclusions

Our current study verified the safety of both biogenic (obtained from nettle) and pyrogenic silica nanoparticles (both below 20 nm grain size) and identified the main mechanism of cell death induced by the tested nanomaterials. On the basis of our findings, it is safe to state that the exposure of silica nanoparticles to HMEC-1 cells causes apoptosis, which is a dominant form of cell death. The usage of nanoparticles initiates oxidative stress, which, in turn, can induce autophagy, apoptosis, and necrosis in a dose-, time-, and size-dependent manner. Taking everything into account, a 24 h period of incubation and a 50 μg/mL concentration of the nanoparticles both allow for their use in further studies, which should be focused on their practical use as therapeutic tools.

## Figures and Tables

**Table 1:** The effect of the silica nanoparticles on the cell cycle. The percentage of the distribution of the G1, S, and G2 stages of the HMEC-1 cell cycle after 72 h of incubating cells with nanosilica. Statistical evaluation of differences was conducted using the ANOVA I and Tukey’s post hoc tests, at significance levels of *p* < 0.01 (*) with respect to the control.

	G1	S	G2
Control	60.85 ± 1.86	25.91 ± 2.04	13.25 ± 0.55
*bio*SiO_2_ 20 µg/mL	61.94 ± 0.62	24.58 ± 0.18	13.49 ± 0.78
*bio*SiO_2_ 50 µg/mL	61.00 ± 0.14	25.49 ± 0.12	13.52 ± 0.02
*bio*SiO_2_ 100 µg/mL	61.81 ± 0.13	25.01 ± 0.33	13.19 ± 0.19
*bio*SiO_2_ 200 µg/mL	58.96 ± 1.56	27.92 ± 1.68	13.12 ± 0.11
Control	48.83 ± 1.33	32.49 ± 1.17	18.69 ± 0.92
*pyr*SiO_2_ 20 µg/mL	47.97 ± 1.22	31.87 ± 0.78	20.16 ± 1.34
*pyr*SiO_2_ 50 µg/mL	48.08 ± 0.91	32.47 ± 0.62	19.44 ± 0.93
*pyr*SiO_2_ 100 µg/mL	48.74 ± 1.89	30.85 ± 0.54	20.41 ± 1.50
*pyr*SiO_2_ 200 µg/mL	49.47 ± 1.14	29.58 ± 0.79 (*)	20.95 ± 1.56 (*)

## Data Availability

Data sharing is not applicable to this article.
